# Magnetoencephalographic Imaging of Auditory and Somatosensory Cortical Responses in Children with Autism and Sensory Processing Dysfunction

**DOI:** 10.3389/fnhum.2017.00259

**Published:** 2017-05-26

**Authors:** Carly Demopoulos, Nina Yu, Jennifer Tripp, Nayara Mota, Anne N. Brandes-Aitken, Shivani S. Desai, Susanna S. Hill, Ashley D. Antovich, Julia Harris, Susanne Honma, Danielle Mizuiri, Srikantan S. Nagarajan, Elysa J. Marco

**Affiliations:** ^1^Department of Radiology, University of California, San FranciscoSan Francisco, CA, United States; ^2^Department of Neurology, University of California, San FranciscoSan Francisco, CA, United States; ^3^Department of Pediatrics, University of California, San FranciscoSan Francisco, CA, United States; ^4^Department of Psychiatry, University of California, San FranciscoSan Francisco, CA, United States

**Keywords:** autism spectrum disorders (ASD), sensory processing disorder, somatosensory evoked fields, auditory evoked fields, processing speed, communication

## Abstract

This study compared magnetoencephalographic (MEG) imaging-derived indices of auditory and somatosensory cortical processing in children aged 8–12 years with autism spectrum disorder (ASD; *N* = 18), those with sensory processing dysfunction (SPD; *N* = 13) who do not meet ASD criteria, and typically developing control (TDC; *N* = 19) participants. The magnitude of responses to both auditory and tactile stimulation was comparable across all three groups; however, the M200 latency response from the left auditory cortex was significantly delayed in the ASD group relative to both the TDC and SPD groups, whereas the somatosensory response of the ASD group was only delayed relative to TDC participants. The SPD group did not significantly differ from either group in terms of somatosensory latency, suggesting that participants with SPD may have an intermediate phenotype between ASD and TDC with regard to somatosensory processing. For the ASD group, correlation analyses indicated that the left M200 latency delay was significantly associated with performance on the WISC-IV Verbal Comprehension Index as well as the DSTP Acoustic-Linguistic index. Further, these cortical auditory response delays were not associated with somatosensory cortical response delays or cognitive processing speed in the ASD group, suggesting that auditory delays in ASD are domain specific rather than associated with generalized processing delays. The specificity of these auditory delays to the ASD group, in addition to their correlation with verbal abilities, suggests that auditory sensory dysfunction may be implicated in communication symptoms in ASD, motivating further research aimed at understanding the impact of sensory dysfunction on the developing brain.

## Introduction

Sensory dysfunction is highly prevalent (≥70%) in Autism Spectrum Disorders (ASD; Greenspan and Wieder, 1997; Mayes and Calhoun, 1999; Adamson et al., 2006; Tomcheck and Dunn, 2007; Al-Heizan et al., 2015), and is associated with autism symptom severity, family stress, and distress, and impairment in communication and socialization (Rogers et al., 2003; Adamson et al., 2006; Brock et al., 2012; Ben-Sasson et al., 2013; Brandwein et al., 2015; Demopoulos et al., 2015a,b; Sanz-Cervera et al., 2015; Corbett et al., 2016). Nevertheless, sensory dysfunction has been acknowledged only recently as a core diagnostic feature of ASD (American Psychiatric Association, 2013). Consequently, our understanding of its neuropathology, both in ASD as well as in children who do not meet ASD criteria, is still emerging. While this dysfunction can manifest in any sensory domain or as deficits in multimodal integration (Khalfa et al., 2004; Rogers and Ozonoff, 2005; Ben-Sasson et al., 2009; Marco et al., 2011; Stevenson et al., 2014), recent evidence suggests that auditory and tactile processing may be among the most severely impacted (Fernandez-Andres et al., 2015). As such, these sensory modalities are the focus of the present study.

### Auditory processing in ASD

Abnormalities in auditory processing are well documented in individuals with ASD (Klin, 1993; Hitoglou et al., 2010; Demopoulos and Lewine, 2016), including auditory filtering deficits (Alcántara et al., 2004; DePape et al., 2012; Tomchek et al., 2014) as well as both impaired (Kargas et al., 2015) and superior pitch perception (Bonnel et al., 2003; Heaton, 2003, 2005; O'Riordan and Passetti, 2006; Mayer et al., 2014; Stewart et al., 2015). There is also substantial evidence of abnormalities in subcortical (Russo et al., 2008, 2009a) and cortical auditory processing, including absent signals, anomalous oscillatory profiles, reduced mismatch signals, impaired rapid processing, and delayed processing components (Gage et al., 2003a,b; Tecchio et al., 2003; Oram Cardy et al., 2005a,b, 2008; Järvinen-Pasley and Heaton, 2007; Tomcheck and Dunn, 2007; Wilson et al., 2007; Russo et al., 2009b; Schmidt et al., 2009; Gandal et al., 2010; Roberts et al., 2010, 2011; Rojas et al., 2011; Edgar et al., 2013, 2014, 2015; Abdeltawwab and Baz, 2015; Demopoulos et al., 2015b).

It is unclear, however, whether the well-documented delays in auditory processing (Gage et al., 2003a,b; Oram Cardy et al., 2005a,b, 2008; Russo et al., 2009b; Gandal et al., 2010; Roberts et al., 2010, 2011; Edgar et al., 2013, 2014, 2015; Abdeltawwab and Baz, 2015; Demopoulos et al., 2015b) are specific to auditory cortex or whether they are part of a more general delay in speed of information processing. For example, there is some evidence of reduced processing speed in ASD (Hedvall et al., 2013; Travers et al., 2014), which has been associated with white matter structural integrity (Lazar et al., 2014; Travers et al., 2014), frontal lobe volume (Schmitz et al., 2007), communication (Oliveras-Rentas et al., 2012), and adaptive functioning (Hedvall et al., 2013). Other studies, however, have failed to identify significant differences in processing speed for participants with ASD relative to typically developing peers (Scheuffgen et al., 2000; Wallace et al., 2009), particularly when motor demands are minimized (Kenworthy et al., 2013). It is likely that these discrepant findings are associated with heterogeneity across the autism spectrum, as one study reported significantly slower processing speed in participants with ASD and a history of delayed speech onset compared to those without a history of speech delay (Barbeau et al., 2015). Notably, assessment of processing speed in these studies employed tasks that evaluate speed of processing visual information rather than auditory information. Thus, in the extant literature, there is evidence to suggest early auditory processing delays in children with ASD; however, the extent to which these delays are domain specific vs. an artifact of a more global processing delay also has yet to be determined.

### Tactile processing in ASD

Studies investigating detection of vibrostimulation in ASD have produced mixed findings, with reports of no differences in tactile detection for individuals with ASD (Güçlü et al., 2007) as well as both raised (Puts et al., 2014) and reduced detection thresholds (Blakemore et al., 2006; Cascio et al., 2008). In studies examining static tactile detection thresholds, differences were not identified between ASD and control groups (O'Riordan and Passetti, 2006; Cascio et al., 2008; Demopoulos et al., 2015a), although reduced variance in task performance suggests that more sensitive measures may be needed to detect subtle differences (Demopoulos et al., 2015a). Some evidence suggests that while groups may not differ in thermal detection, individuals with ASD may experience increased thermal pain (Cascio et al., 2008). Other studies have reported greater and more variable perception of surface roughness (Haigh et al., 2015) as well as impairments in stereognosis in ASD (Abu-Dahab et al., 2013). In our prior work examining performance on a battery of tactile measures, including tactile detection, sensitivity, form discrimination, and proprioception, only tactile proprioception was significantly impaired in the ASD group relative to typically developing control (TDC) participants (Demopoulos et al., 2015a).

Neuroimaging studies of cortical somatosensory response to tactile stimulation have identified abnormalities in tactile sensory processing and even neural organization of the somatosensory cortex (Coskun et al., 2009). For example, reduced amplitude of the magnetoencephalography (MEG) somatosensory evoked response to tactile stimulation has been identified in ASD, and was significantly associated with parent-reported tactile dysfunction (Marco et al., 2012). This is consistent with one prior study that demonstrated reduced BOLD fMRI response to tactile stimulation in the primary somatosensory cortex (Cascio et al., 2012), although another fMRI study reported enhanced somatosensory response to tactile stimulation in ASD participants (Kaiser et al., 2015). Structural abnormalities also have been related to tactile sensory behavior in ASD. Specifically, increased tactile defensiveness has been associated with reduced fractional anisotropy in the inferior longitudinal fasciculus (Pryweller et al., 2014). Thus, the weight of the evidence suggests there are increased rates of tactile dysfunction in individuals with ASD, although the degree and nature of this dysfunction may vary for each affected individual.

### Sensory processing dysfunction in the absence of an ASD

It is estimated that >5% of children experience sensory processing dysfunction (SPD) who do not demonstrate the primary language or social deficits sufficient to meet criteria for an ASD diagnosis (Ahn et al., 2004). Despite the substantial impairment that SPD can cause in social and adaptive functioning, a lack of a clinical diagnostic label often leaves these children without access to resources for treatment. Yet, prior work suggests that there are measurable biological differences, such as white matter abnormalities, in children with SPD (Owen et al., 2013; Chang et al., 2014), that these biological differences are associated with sensory processing behavior (Chang et al., 2016), and that their profile of sensory dysfunction may demonstrate important distinctions from the presentation of sensory dysfunction in ASD (Demopoulos et al., 2015a).

### Rationale and hypotheses

The present study examined cortical auditory and somatosensory evoked fields as well as performance-based measures of auditory and tactile sensory processing in ASD, SPD, and TDC groups. Concurrent examination of these groups provides an opportunity to identify aspects of cortical sensory dysfunction that are associated with other ASD symptomatology (e.g., social and communication deficits), as well as to better understand children with SPD who are understudied and have limited access to services despite their significant functional impairment (Carter et al., 2011; Ben-Sasson et al., 2013; Gourley et al., 2013). Prior evidence suggests that somatosensory response amplitude in ASD is reduced (Cascio et al., 2012) and is associated with tactile dysfunction (Marco et al., 2012). Impaired tactile performance has also been demonstrated in both ASD and SPD groups relative to TDC participants (Demopoulos et al., 2015a). Based on these preliminary findings, we hypothesized that (1) somatosensory response amplitudes would be reduced in the ASD and SPD groups relative to TDC participants. We also hypothesized that these results would extend to response latency, and that the results would follow the same pattern identified in our prior work concurrently examining these three groups on behavioral measures. Specifically, based on well established evidence of delayed auditory latency in ASD (Gage et al., 2003a,b; Oram Cardy et al., 2005a,b, 2008; Russo et al., 2009b; Gandal et al., 2010; Roberts et al., 2010, 2011; Edgar et al., 2013, 2014, 2015; Abdeltawwab and Baz, 2015; Demopoulos et al., 2015b), and our behavioral evidence that while tactile dysfunction was shared in groups with ASD and SPD, auditory deficits were specific to ASD, we hypothesized that (2) the ASD group would show auditory and somatosensory response delays, whereas, the SPD group would only show somatosensory response delays. Likewise, based on prior evidence of associations between cortical auditory response abnormalities and communication deficits in ASD (Oram Cardy et al., 2005a; Russo et al., 2009b; Schmidt et al., 2009; Roberts et al., 2011; Edgar et al., 2013), we hypothesized that (3) cortical auditory response differences would be associated with poor communication in ASD participants. Because prior work identified somatosensory response abnormalities in ASD that were associated with tactile dysfunction (Marco et al., 2012), and because impaired tactile performance has also been demonstrated in both ASD and SPD groups relative to TDC participants (Demopoulos et al., 2015a), we also hypothesized that (4) somatosensory response differences would be associated with performance on measures of tactile functioning in the ASD and SPD groups. Finally, it has yet to be determined whether the well-established delays in cortical auditory response latency in ASD reflect a specific deficit in auditory processing vs. a generalized delay in speed of processing across domains of functioning. The extant literature has demonstrated mixed findings on processing speed (measured via visual tasks) in ASD (Scheuffgen et al., 2000; Wallace et al., 2009; Hedvall et al., 2013; Kenworthy et al., 2013; Travers et al., 2014), and it is unclear if those with cortical auditory response delays are the same individuals who demonstrate slower processing speed in these studies. Given the specific domains of functioning associated with auditory processing that are impacted in ASD (e.g., communication impairments, sound sensitivities, etc.), we hypothesized that (5) delays in cortical auditory response latency in ASD would be domain-specific, and would not be associated with processing speed deficits or cortical response delays in another sensory domain (i.e., on the somatosensory processing task).

## Methods

### Participants

Participants were 50 boys (ASD *N* = 18, SPD *N* = 13, and TDC *N* = 19) ages 8–12, who were recruited from the UCSF Sensory Neurodevelopmental and Autism Program (SNAP) participant registry and website, UCSF SNAP clinic, and local online parent groups. All participants who were taking medication were on a stable dose for at least 6 weeks prior to testing. For the TDC group, one participant regularly used an antihistamine and a leukotriene inhibitor for seasonal allergies as well as melatonin for sleep. In the SPD group, one participant was prescribed lisdexamfetamine, sertraline, and valproic acid for inattention and challenging behavior and two others were taking stimulants (amphetamine/dextroamphetamine and methylphenidate) for inattention. In the ASD group, one participant was receiving a chelation agent (DMSA), another was taking escitalopram for anxiety, and a third participant was taking guanfacine and methylphenidate for calming and inattention.

Exclusion criteria were brain malformation or injury, movement disorder, bipolar disorder, psychotic disorder, hearing impairment, or Wechsler Intelligence Scale for Children-Fourth Edition (WISC-IV; Wechsler, 2003) Perceptual Reasoning Index (PRI) score <70. The Social Communication Questionnaire (SCQ; Rutter et al., 2003) and the Sensory Profile (Dunn, 1999) were administered for all participants. Those scoring ≥15 on the SCQ or who had a prior clinical diagnosis of ASD were evaluated with the Autism Diagnostic Inventory-Revised (ADI-R; Lord et al., 1994) and the Autism Diagnostic Observation Schedule (ADOS; Lord et al., 1989). Participants in the ASD group met diagnostic cutoffs on both of these measures and met DSM-IV-TR criteria for Autistic Disorder, confirmed by a pediatric neurologist (EJM). Participants assigned to the SPD group had been previously diagnosed with SPD by a community occupational therapist. Inclusion criteria for this group were SCQ < 15 and a score in the “Definite Difference” range (<2% probability) in one or more domains of the Sensory Profile, including auditory, visual, oral/olfactory, tactile, vestibular, or multisensory processing. Participants in the TDC group did not score in any clinical ranges on the SCQ or Sensory Profile. Table 1 presents demographic information.

**Table 1:** **Group characteristics (M ± SD [range])**.

	**ASD**	**SPD**	**TDC**	**Statistics**
Age	9.82 ± 1.17 [8.13–11.80]	9.27 ± 0.95 [8.12–11.94]	9.79 ± 1.11 [8.18–11.45]	*F*_(2, 47)_ = 1.15
VCI	101.89 ± 18.08 [65–140]^b^^d^	115.69 ± 11.38 [99–136]	117.42 ± 12.85 [93–138]	*F*_(2, 47)_ = 5.96^**^
PRI	101.56 ± 13.37 [71–131]^b^^c^	117.23 ± 10.49 [94–131]	114.00 ± 12.26 [92–133]	*F*_(2, 47)_ = 7.52^**^
PSI	87.00 ± 11.73 [65–109]^b^	95.77 ± 15.00 [70–118]	100.53 ± 13.17 [75–128]	*F*_(2, 47)_ = 4.57^*^
**SENSORY PROFILE DOMAIN SCORES**				
Auditory	24.72 ± 6.037 [13–33]^a^	21.08 ± 7.57 [12–36]	33.95 ± 3.05 [28–40]^e^	*F*_(2, 47)_ = 23.33^**^
Visual	35.17 ± 7.59 [14–45]^b^	31.62 ± 5.28 [25–40]	40.84 ± 2.93 [35–45]^e^	*F*_(2, 47)_ = 11.20^**^
Vestibular	46.83 ± 4.77 [37–54]^b^^d^	43.38 ± 6.41 [34–55]	51.63 ± 2.52 [45–55]^e^	*F*_(2, 47)_ = 12.98^**^
Touch	70.11 ± 11.36 [44–87]^b^^d^	62.69 ± 13.12 [40–88]	82.47 ± 5.35 [70–90]^e^	*F*_(2, 47)_ = 15.93^**^
Multisensory	24.56 ± 3.45 [17–30]^b^	21.69 ± 6.76 [10–33]	31.00 ± 2.69 [25–35]^e^	*F*_(2, 47)_ = 20.01^**^
Oral	45.56 ± 10.14 [31–59]^f^	41.92 ± 12.04 [23–59]	52.84 ± 8.23 [32–60]^c^	*F*_(2, 47)_ = 5.06^*^
**ETHNICITY (N)**				
Caucasian	11	7	11	
Asian	2	1	0	
Multiracial	5	4	3	
Unknown	0	1	5	
**HANDEDNESS**				
Right	15	12	16	
Left	1	0	0	
Ambidextrous	2	0	0	
Unknown	0	1	3	

* *p ≤ 0.01*.

** *p ≤ 0.001*.

a *Significantly different from TDC at p < 0.001*.

b *Significantly different from TDC at p < 0.01*.

c *Significantly different from SPD at p < 0.01*.

d *Significantly different from SPD at p < 0.05*.

e *Significantly different from SPD at p < 0.001*.

f *Significantly different from TDC at p < 0.05*.

### Measures

#### Intelligence and processing speed

Indices of Verbal Comprehension (VCI), Perceptual Reasoning (PRI), and Processing Speed (PSI) were assessed on the WISC-IV (Wechsler, 2003). Because communication impairments are a diagnostic feature of ASD as well as an outcome measure of interest, the PRI rather than the VCI or FSIQ was utilized as a study exclusion measure. The PRI has been shown to be a valid and reliable measure of nonverbal IQ with internal consistency reliability coefficients ranging from 0.91 to 0.93 and stability coefficients ranging from 0.81 to 0.87 (Sattler and Dumont, 2004).

#### Communication

The Acoustic-Linguistic Index (ALI) of the Differential Screening Test for Processing (DSTP; Richard and Ferre, 2006) was used to assess auditory processing skills associated with language. This index is comprised of subtests assessing phonic and phonemic manipulation. The Linguistic Index (LI) of the DSTP, comprised of subtests evaluating knowledge of antonyms, prosodic interpretation, and language organization, was used to assess semantic and pragmatic aspects of language. The VCI of the WISC-IV (Wechsler, 2003) was administered to index general verbal intellectual abilities.

#### Tactile processing

Tactile form discrimination was assessed using the Von Boven Domes task (Von Boven Domes, 2018), for which a series of plastic domes are pressed against the left index fingertip one at a time with 100gr_f_force for 1 s each. Each dome has a different grating, spaced at 3.0, 2.0, 1.5, 1.2, 1.0, 0.75, 0.5, and 0.35 mm, respectively. Beginning with the 3.0 mm ridge, participants stated whether the ridge was aligned “along” or “across” the finger. Each trial consisted of 10 vertical or horizontal orientations presented in a standardized unpredictable order. Grating spacing was progressively lowered at each trial, and after three errors were made in a trial, testing was discontinued. Form discrimination was quantified by the lowest grating size (mm) trial passed.

Finally, the graphesthesia subtest of the Sensory Integration Praxis Tests (Ayres, 1989) measured *tactile proprioception* by asking participants to recreate seven designs (neither numbers nor letters) drawn on the dorsum of each hand with closed eyes. Drawings were scored 0–2 for accuracy and totaled for each hand as measures of left- and right-handed tactile proprioception.

#### Magnetoencephalography

Auditory and somatosensory evoked fields were assessed for the 500 standard (non-oddball) trials of passive mismatch field tasks. For the auditory task, participants passively listened to a 1000 Hz tone burst with a 5 ms ramp of either 50 ms (for the standard stimulus condition, 500 trials) or 100 ms duration (deviant condition, 100 trials) presented at random through MEG compatible headphones at 65 dB monaurally to the right ear only. The delay resulting from the sound traveling through the headphones was minimal (3.2 ms) and consistent across participants. The interstimulus interval was 870 ms with a 100 ms jitter range. For somatosensory evoked fields tactile stimulation was administered via application of a 17 pounds per square inch (PSI) tap via a balloon diaphragm (4D Neuroimaging/Biomagnetic Technologies)^1^ to the left middle (LD3 for the standard stimulus condition, 500 trials) or index fingertip (LD2 for deviant condition, 100 trials) at random with the same proportions of standard to deviant trials as the auditory task and an interstimulus interval of 800 ms with a 100 ms jitter range.

### Procedures

Informed consent was obtained from parents or legal guardians and participants who were 13 years old, with assent of all participants under age 13 in accordance with the UCSF Institutional Review Board protocol. Participants completed the diagnostic, behavioral, MEG, and MRI exams at three separate testing appointments. MEG data were collected at the UCSF Biomagnetic Imaging Laboratory using a 275-channel CTF System whole-head biomagnetometer (MEG International Services Ltd., Coqiotlam, BC, Canada) at a 1200 Hz sampling rate. Localization coils were placed at the nasion and bilateral peri-auricular points. During the scan participants laid in a supine position with their heads in a one inch foam padded MEG helmet to minimize head motion and to standardize the distance between the top of the head and the sensor array, allowing for optimal comparisons of response amplitudes between participants of different head sizes.

#### MEG data processing

Baseline correction and artifact removal were performed prior to averaging. Specifically, sensor data > 2pT were identified and visually inspected for artifact (including muscle, eye blink, and motion). ANOVA results comparing numbers of artifact-free trials for the auditory evoked fields indicated that groups did not significantly differ in number of trials, *F*_(2, 45)_ = 1.94, *p* = 0.155; however, the data from TDC group had significantly more artifact-free trials that the ASD or SPD groups for somatosensory evoked fields, *F*_(2, 46)_ = 6.07, *p* = 0.005 (Table 2). Despite these differences all groups had a sufficiently large number of somatosensory trials remaining (≥406).

**Table 2:** **Number of trials remaining after artifact rejection for auditory and somatosensory evoked response tasks**.

**Measure**	**Study Group**			***F***	**Partial η^2^**
	**ASD M ± SD [Range]**	**SPD M ± SD [Range]**	**TDC M ± SD [Range]**		
**AUDITORY EVOKED FIELDS (500 TRIALS PRESENTED)**					
Artifact free trials	446.67 ± 26.52 [349–475]	438.08 ± 43.23 [360–490]	461.44 ± 31.47 [377–492]	1.94	0.08
**SOMATOSENSORY EVOKED FIELDS (500 TRIALS PRESENTED)**					
Artifact free trials	459.83 ± 19.31 [406–489]	452.92 ± 21.16 [416–488]	475.78 ± 17.11^a^^b^[438–496]	6.07^*^	0.21

* *p = 0.005*.

a *Significantly different from ASD group at p < 0.05*.

b *Significantly different from SPD group at p < 0.01*.

For the purposes of this study (examining basic unimodal auditory and somatosensory response) only the standard conditions of the MEG tasks were averaged in order to maximize signal to noise ratio. Raw data from all sensors were coregistered to the participant's own T1-weighted 3T MRI, averaged, and passed through the Champagne source reconstruction algorithm (Owen et al., 2012a,b) over the 25–400 ms post-stimulus interval. The Champagne algorithm assumes a dipole for each orientation in each voxel whose time-course is estimated from data. From this a single time-course is estimated calculating root-mean square time-course from the source orientations. Unfiltered data is used at this stage of data processing to better estimate the signal and noise covariances that are used in our source reconstruction algorithm. The champagne algorithm results in a whole brain source reconstruction and not only reconstructs correlated source with high spatial resolution, it also reconstructs source time-courses (i.e., brain activity) with significantly higher accuracy than any other benchmark algorithms including MNE (e.g., see Owen et al., 2012b for demonstration of a consistent and robust reconstruction of auditory cortex using Champagne). These results were then used to localize the maximum activation in the right somatosensory cortex over a 30–80 ms window for the somatosensory evoked field. For the auditory localization a time window of 80–180 ms was used to identify the voxel with maximum activation of the M100 response over the auditory cortex. Raw data was then band-pass filtered (2–40 Hz) before the activation weights of the identified source voxel were applied to the waveform and then averaged. Finite impulse response filters and were applied to data with no phase distortion, therefore filtering did not impact any of our latencies.

**Figure 1:**
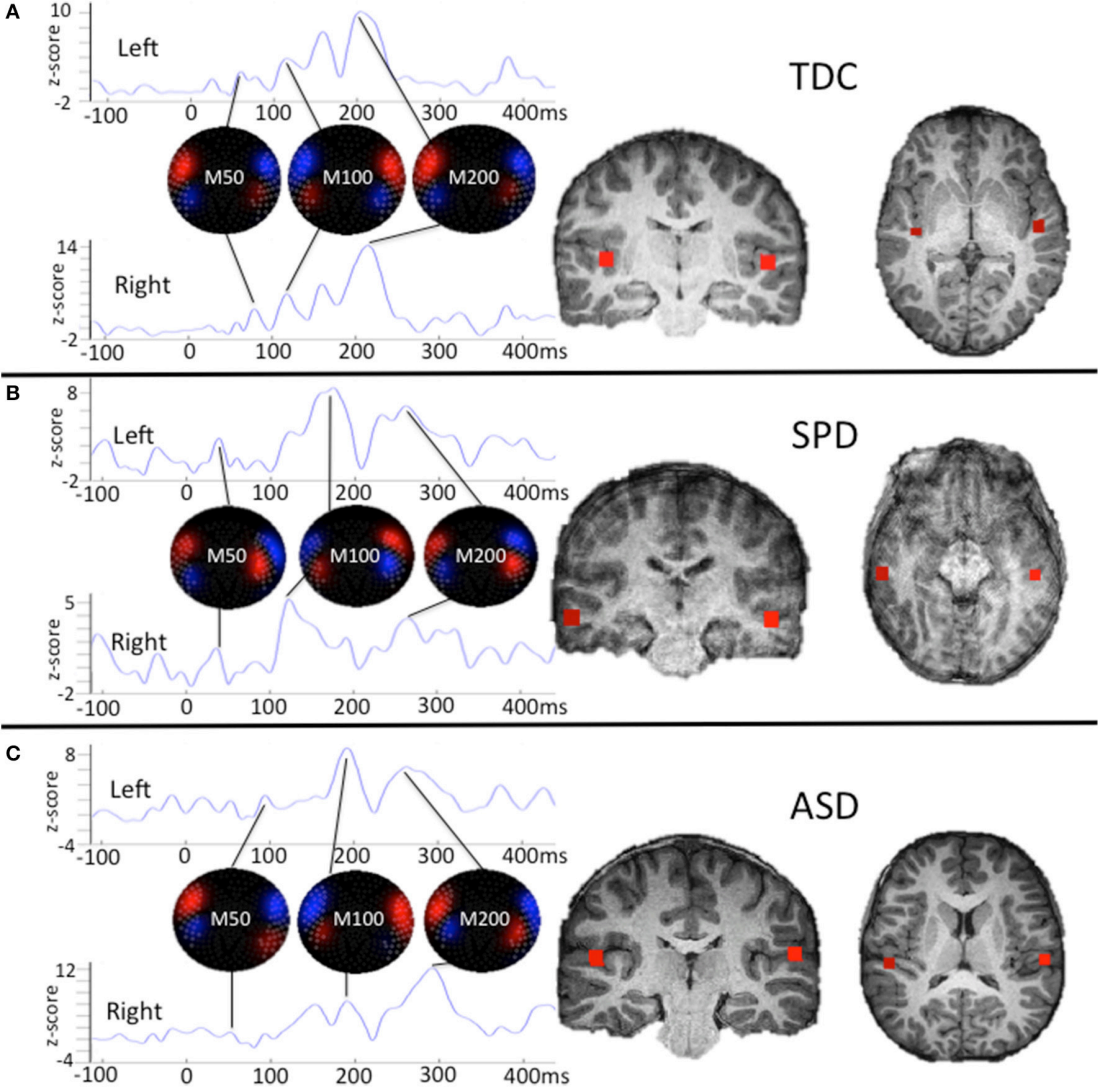
**Single participant examples of auditory evoked field source localizations and corresponding waveforms with flux topography for each peak**. The waveform amplitude is normalized within subject on the y axis, which represents the z-score of the source waveform amplitude based on the pre-stimulus baseline (originally measured in fT). The timescale on the horizontal axis is in milliseconds. For **(A)**, the chronology of changing flux topography identifies peaks for the M50, M100, and M200 that roughly correspond to their expected latencies in a TDC participant. For **(B)**, the SPD participant demonstrates delays in the right M200 and left M100 and M200 responses. For **(C)**, the ASD participant demonstrates a weak right M50 response and delays for all other responses. This profile of responses for these three participants parallels findings from group level analyses, with the SPD group demonstrating an intermediate phenotype between the auditory evoked responses for the ASD and TDC groups.

**Figure 2:**
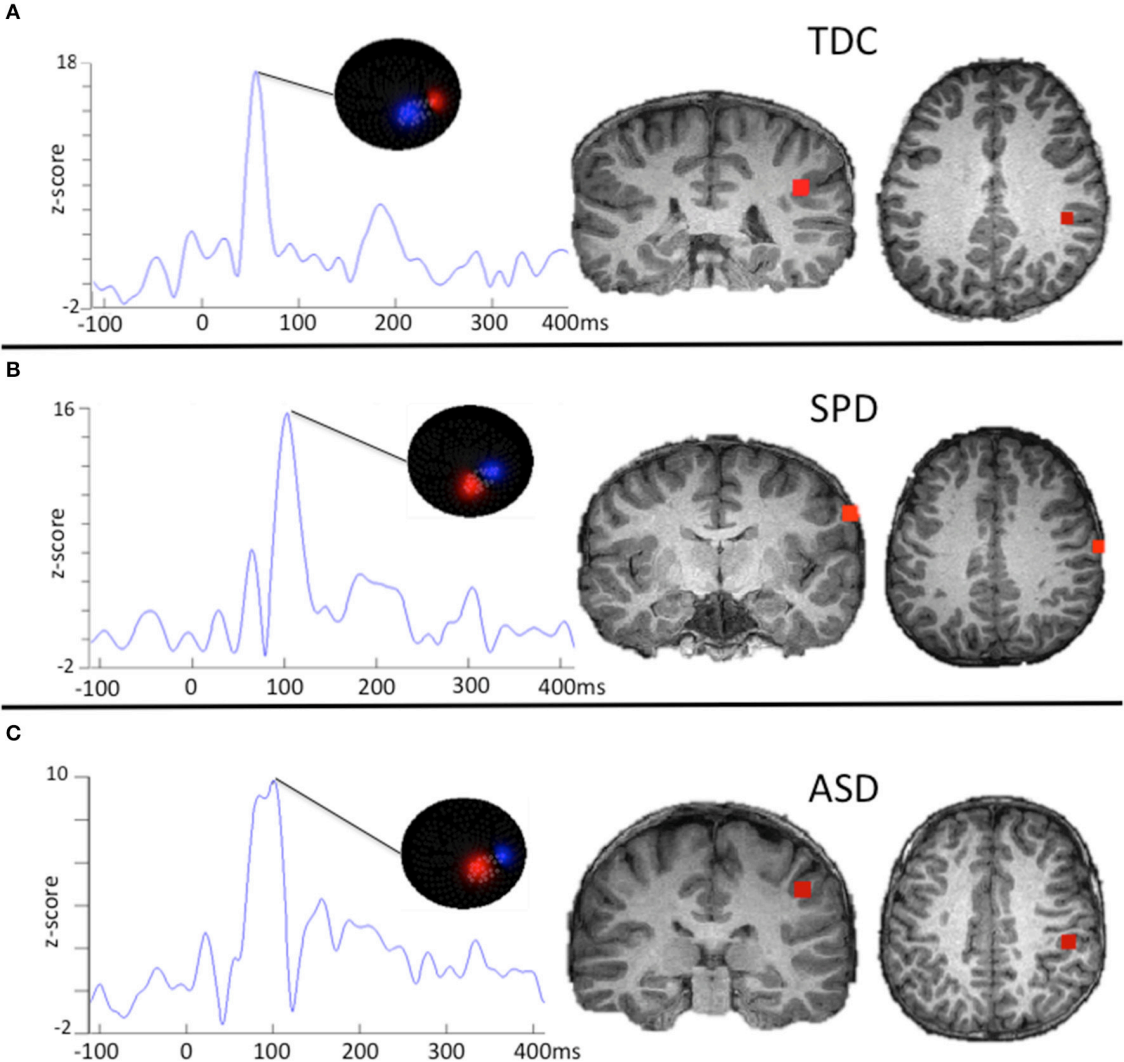
**Examples of somatosensory evoked fields source localizations and corresponding waveforms with flux topography**. The waveform amplitude is normalized within subject on the y axis, which represents the z-score of the source waveform amplitude based on the pre-stimulus baseline (originally measured in fT). The timescale on the horizontal axis is in milliseconds. For **(A)**, the flux topography identifies the Somatosensory (SS) peak that roughly corresponds to the expected latency in TDC participants. For **(B)**, the SPD participant demonstrates a delayed SS latency that does not meet a stringent significance threshold relative to the TDC or ASD cohort. For **(C)**, the ASD participant demonstrates a delayed SS response that is characteristic for the group as a whole.

Variables of interest for tactile processing were the right (contralateral) somatosensory evoked field amplitude and latency. Variables of interest for auditory processing were the bilateral M50, M100, and M200 auditory evoked field amplitudes and latencies. Although a time window of 80–180 ms was used for auditory source localization, individual M50, M100, and M200 peaks of the resultant waveforms were not identified based on strict temporal cutoffs, but rather, a combination of their amplitude, latency, and the chronology of their flux topographies (Edgar et al., 2014) manually identified across the 25–400 ms waveform. This decision was based on prior work identifying response delays in auditory evoked fields of children with ASD (Oram Cardy et al., 2005b; Gandal et al., 2010; Roberts et al., 2010, 2011; Edgar et al., 2014; Demopoulos et al., 2015b). Specifically, the M100 response was classified as the peak preceded and followed by a peak of opposite flux topography (the M50 and M200, respectively). These peaks would correspond to EEG P50, N100, and P200 responses (see Ross and Tremblay, 2009, for a discussion of the role of the N100 and P200 in repeated auditory stimulus exposure). If no M50 response could be clearly identified then the M100 was classified as the peak preceding an M200 (with opposite flux topography). A second blinded manual scoring of peaks was performed for the auditory M100 and M200 (M50 was not included in this analysis because M50 peaks were absent in so many participants). Reliability analysis between raters for peak assignment resulted in an 84% agreement. Examples of source localizations with corresponding waveforms and flux topography from a participant in each group are presented in Figure 1 for auditory evoked fields and in Figure 2 for somatosensory evoked fields.

### Data analytic plan

To test the hypotheses (1 and 2) that groups differ in their cortical auditory and somatosensory response amplitudes and latencies, the following analyses were performed. First, because a substantial number of participants did not produce an identifiable M50 auditory response, z-tests of independent proportions were performed to determine if rates of missing M50 responses differed between groups. Next, because age has been associated with the latency of cortical auditory response (Gage et al., 2003b), analyses of covariance (ANCOVAs) were performed to determine if groups differed in the latency or amplitude of the M100 or M200 auditory response in each hemisphere after controlling for age. These ANCOVAs were also performed for the latency and amplitude of the right hemisphere (contralateral) somatosensory response. *Post-hoc* independent samples *t*-tests were performed when ANCOVA results achieved statistical significance to determine which groups significantly differed from each other.

Then, given the substantial heterogeneity in language abilities across individuals on the autism spectrum, we sought to test the hypothesis (3) that significant differences in cortical auditory processing would be associated with communication in ASD but not SPD or TDC participants. To do this, we performed correlations between performance on communication measures (ALI, LI, and VCI) and cortical auditory response deficits identified via statistically significant ANCOVA results. We also assessed the specificity of associations between these auditory response deficits and communication by also examining correlations with nonverbal cognitive abilities via the Perceptual Reasoning Index (PRI) of the WISC-IV and performing a hierarchical regression analysis with PRI entered at Step 1, communication measures entered at Step 2, and with cortical auditory delay as the dependent variable. Correlations were also performed between these cortical auditory responses and the Auditory scale of the Sensory Profile. Likewise, correlations were performed between cortical somatosensory response deficits and tactile performance measures as well as the Tactile scale of the Sensory Profile (Hypothesis 4). Finally, to test the hypothesis (5) that cortical response latency delays are domain specific rather than a reflection of general processing delays, correlations were performed between auditory and tactile cortical response deficits, as well as between cortical response deficits and cognitive processing speed (PSI) to determine if these delays were associated with one another.

## Results

A reliable auditory source localization could not be obtained for one TDC participant, who was therefore not included in analyses of cortical auditory response; however, somatosensory data was able to be used for this participant. The proportion of missing auditory M50 responses did not differ between groups. For the left hemisphere, an identifiable M50 auditory response could not be detected in 30% of SPD participants and 38.9% of TDC and ASD participants (*z* = 0.310, *p* = 0.757). For the right hemisphere, 44% of participants with ASD did not produce an identifiable M50 response compared to 38.9% in the TDC group and 41.7% in the SPD group (ASD vs. TDC *z* = 0.311, *p* = 0.756, SPD vs. TDC *z* = 0.135, *p* = 0.892, ASD vs. SPD *z* = 0.143, *p* = 0.887).

**Figure 3:**
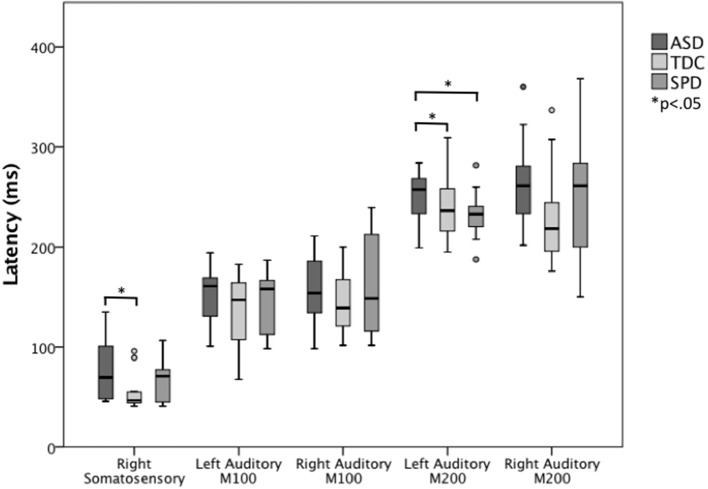
**Somatosensory and auditory response latencies across groups**. The left auditory M200 response is significantly delayed in the ASD group relative to both the SPD and TDC groups, whereas the ASD somatosensory response is significantly delayed relative to only the TDC group.

Next, ANCOVAs were performed to determine if groups differed in the latency or amplitude of the M100 or M200 auditory response in each hemisphere, or the contralateral somatosensory response, after controlling for age (Table 3). Response amplitudes did not differ between groups; however, significant group differences were identified for the left auditory M200 latency, *F*_(2, 45)_ = 3.61, *p* = 0.035, and right somatosensory latency *F*_(2, 46)_ = 3.63, *p* = 0.035. Specifically, the ASD group's left M200 latency was significantly delayed relative to both the TDC and SPD groups, whereas the somatosensory response in the ASD group was only delayed relative to the TDC group and did not significantly differ from the SPD group, who presented with an intermediate somatosensory latency (Figure 3). Likewise, response latencies for the M100 response followed a similar pattern to the M200, with the clinical groups showing longer mean latencies bilaterally, although group differences were not statistically significant.

**Table 3:** **Auditory and somatosensory cortical responses (Raw Scores and ANCOVA Results)**.

**Measure**	**Study Group**			***F***	**Partial η^2^**
	**ASD M ± SD [Range]**	**SPD M ± SD [Range]**	**TDC M ± SD [Range]**		
**LATENCIES (MS)**					
Left M100	153.86 ± 29.20 [100.80–194.20]	145.64 ± 30.83 [98.33–186.70]	135.31 ± 32.87 [67.50–182.50]	1.90	0.08
Right M100	157.52 ± 34.34 [98.33–210.80]	160.23 ± 50.91 [101.7–239.2]	144.38 ± 27.71 [101.70–200.00]	0.71	0.03
Left M200	251.43 ± 22.50^a^^*^ [199.20–284.20]	232.23 ± 24.01 [187.50–281.70]	232.06 ± 35.99 [195.00–309.20]	3.61^*^	0.14
Right M200	260.32 ± 42.32 [201.70–360.00]	251.59 ± 61.78 [150.00–368.30]	229.92 ± 44.21 [175.80–336.70]	2.02	0.08
Right Somatosensory	74.58 ± 28.91^b^^*^ [45.83–135.00]	66.09 ± 22.28 [40.83–106.70]	54.08 ± 17.20 [40.83–95.83]	3.63^*^	0.14
**AMPLITUDES (STANDARDIZED)**					
Left M100	7.43 ± 5.45 [2.31–22.31]	5.81 ± 3.93 [0.43–12.96]	5.72 ± 5.45 [3.06–17.22]	0.90	0.04
Right M100	5.89 ± 3.32 [0.66–13.63]	3.77 ± 2.45 [1.38–10.85]	4.92 ± 3.96 [1.71–16.68]	1.16	0.05
Left M200	7.43 ± 3.41 [2.17–12.79]	7.42 ± 4.37 [1.11–17.47]	7.98 ± 5.18 [3.07–20.19]	0.08	0.00
Right M200	5.78 ± 3.50 [0.57–12.18]	4.86 ± 2.65 [1.90–10.30]	7.84 ± 6.69 [2.71–29.31]	1.24	0.05
Right Somatosensory	15.84 ± 5.83 [4.56–29.47]	13.44 ± 6.26 [5.68–26.34]	15.73 ± 8.71 [3.44–39.24]	0.34	0.02

* *p < 0.05*.

a *Significantly different from TDC and SPD groups at p < 0.05*.

b *Significantly different from TDC groups at p < 0.05*.

**Figure 4:**
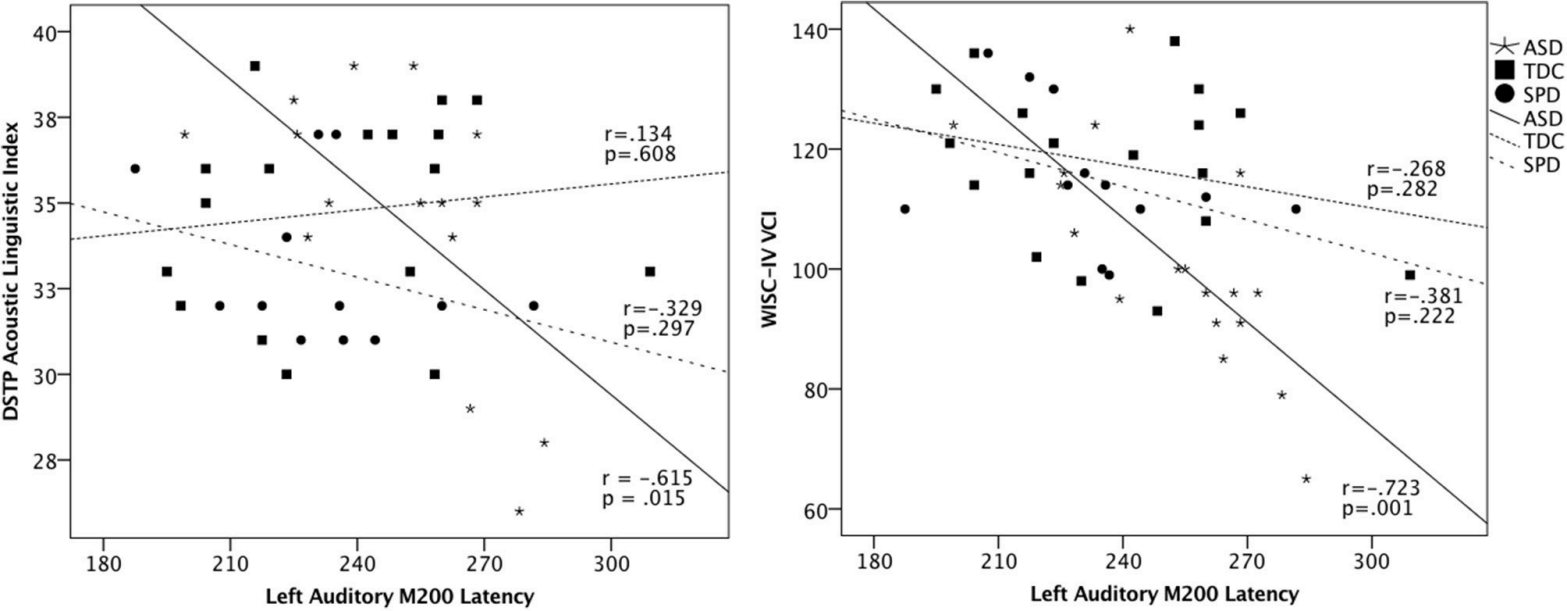
**Scatterplots of DSTP Acoustic Linguistic Index, WISC-IV Verbal Comprehension Index, and the left auditory M200 latencies in each group**. These associations were statistically significant in the ASD group, who showed a significant delay in the M200 response; however, the inclusion of all groups in the scatterplot highlights the intermediate profile of the SPD group relative to the ASD and TDC participants.

To determine whether the left M200 cortical auditory processing delays identified in the ASD group would be associated with communication and parent-reported sensory dysfunction, correlations were performed between the left auditory M200 latency and performance on communication measures and the Auditory scale of the Sensory Profile. Although no significant latency association was found for LI (*r* = −0.371, *p* = 0.130) or the Sensory Profile Auditory scale (*r* = −0.372, *p* = 0.141), significant associations were found between left M200 latency and communication measures, with VCI *r* = −0.723, *p* = 0.001 and ALI *r* = −0.615, *p* = 0.015 (Figure 4). These associations with the left M200 auditory latency appeared to be specific to communication abilities, as the correlation between left M200 and nonverbal cognitive abilities (WISC-IV PRI) was not statistically significant (*r* = −0.202, *p* = 0.422). Further, a hierarchical regression analysis performed with PRI entered in Step 1, communication variables entered in Step 2 (ALI, LI, and VCI), and left M200 latency as the dependent variable demonstrated that communication ability accounted for 60.3% of the variance in left M200 latency after controlling for the effects of nonverbal intelligence (which accounted for 5.7% of the variance) in the ASD group, with *F*_(3, 10)_ = 5.91, *p* = 0.014. These associations were not significant in the SPD or TDC groups (see Table 4). Correlations also were not significant between the somatosensory latency delays identified in the ASD group and the Sensory Profile Tactile scale (*r* = 0.089, *p* = 0.735) or performance on tactile proprioception (left hand *r* = 0.070, *p* = 0.796; right hand *r* = 0.194, *p* = 0.472) and form discrimination tasks (*r* = 0.144, *p* = 0.595). For group statistics on the tactile performance battery see Demopoulos et al. (2015a). Finally, the cortical auditory M200 response latency delays detected in the ASD group were not significantly associated with delayed somatosensory latencies (*r* = 0.172, *p* = 0.494), nor were they associated with PSI (Left M200 *r* = −0.235, *p* = 0.381; somatosensory *r* = −0.344, *p* = 0.192).

**Table 4:** **Group regression models of communication skills on auditory response delays**.

**Model**	**B**	**SeB**	**β**	**Tolerance**	**VIF**
**ASD**					
**Step 1 (R^2^ = 0.06)**					
PRI	−0.39	0.44	−0.24	1.00	1.00
Step 2 (R^2^ change = 0.60)^*^					
PRI	0.20	0.34	0.12	0.78	1.29
ALI	−1.57	1.76	−0.26	0.40	2.52
LI	0.68	1.66	0.10	0.62	1.61
VCI	−1.01	0.36	−0.70^*^	0.55	1.82
**SPD**					
**Step 1 (R^2^ = 0.13)**					
PRI	−0.84	0.67	−0.37	1.00	1.00
**Step 2 (R^2^ change = 0.23)**					
PRI	−0.54	0.79	−0.24	0.76	1.31
ALI	−4.80	3.84	−0.46	0.67	1.50
LI	1.82	2.67	0.30	0.48	2.09
VCI	−1.07	0.80	−0.52	0.60	1.66
**TDC**					
**Step 1 (R^2^ = 0.10)**					
PRI	−0.79	0.60	−0.32	1.00	1.00
**Step 2 (R^2^ change = 0.15)**					
PRI	−1.00	0.68	−0.41	0.83	1.21
ALI	−0.20	2.92	−0.02	0.83	1.20
LI	−3.84	4.31	−0.31	0.51	1.98
VCI	−0.31	0.77	−0.13	0.64	1.57

* *p < 0.05*.

## Discussion

To understand the impact of sensory dysfunction in ASD and more broadly we must examine associations between sensory behavior and brain activity that may cross diagnostic boundaries. Prior to this study, a direct comparison of cortical sensory processing was lacking between children with ASD and those with SPD who do not meet ASD criteria. Thus, the present study examined both performance-based sensory processing and cortical auditory and somatosensory evoked fields in ASD, SPD, and TDC participants.

First, we hypothesized that contralateral somatosensory response amplitudes would be reduced in the ASD and SPD groups relative to TDC participants. This hypothesis (1) was not supported, as we failed to detect group differences in any response amplitudes (auditory or somatosensory). This is in contrast to our prior work identifying reduced somatosensory response amplitudes in ASD (Marco et al., 2012), although these findings were specific to the left hemisphere, which was not assessed in the present study. Our findings are consistent, however, with one prior study in which no significant differences were found between ASD and TDC participants in cortical somatosensory response to tactile stimulation (Cascio et al., 2015), although in this study stimulation was administered to the dominant hand, so findings cannot be interpreted for a specific hemisphere. In our prior study examining group differences on the performance-based tactile battery described in the present study, no significant group differences were detected on measures of left-handed graphesthesia or tactile form discrimination (Demopoulos et al., 2015a). Based on these results, it is not surprising that we also failed to detect group differences in cortical somatosensory response. Given the variability in the clinical presentation of sensory dysfunction for individuals with ASD, these divergent findings may be associated with heterogeneity in somatosensory response across the autism spectrum or may reflect a more robust left hemisphere amplitude effect, which was not assessed in this current study. These null findings may also reflect the lack of power to detect moderate and small effects. Replication with a larger sample is necessary to determine whether the findings in the present study are associated with a failure to replicate prior results vs. insufficient power.

Second, based on our prior study of performance-based measures of sensory processing in these groups (Demopoulos et al., 2015a), we hypothesized (2) that the ASD group would show delays in auditory and somatosensory evoked fields, whereas the SPD group would only show delays for somatosensory evoked fields. These hypotheses were partially supported, as only the ASD group was significantly delayed relative to the TDC group for the contralateral somatosensory response; however, the ASD group demonstrated significantly delayed left M200 auditory response relative to both other groups. Interestingly, group differences in the M100 response did not achieve statistical significance as has been reported in prior ASD studies (Gage et al., 2003b; Gandal et al., 2010; Roberts et al., 2010; Matsuzaki et al., 2012), although our M100 responses followed the same pattern of response latency as the M200, with the longest latencies in the ASD group. Our failure to replicate these significant M100 findings is likely due to a smaller sample size in the present study, limiting our power to detect statistical significance. Consistent with prior work, we also failed to detect group differences in the proportion of missing M50 auditory responses for either hemisphere (Edgar et al., 2014). With regard to lateralization, the left hemisphere M200 response showed the greatest delay, in contrast to previous studies reporting only right hemisphere M100 delays (Edgar et al., 2013, 2014). There is, however, one prior study reporting a trend for longer M100 latencies in the left hemisphere for low frequency tones in ASD compared to TDC participants (Gage et al., 2003b).

Next, we hypothesized (3) that auditory response delays would be associated with communication deficits in ASD. This hypothesis was supported by the data, as the M200 delays identified in the ASD group were significantly associated with scores on the Acoustic-Linguistic Index of the DSTP and the Verbal Comprehension Index of the WISC-IV (Figure 4). We did not hypothesize that these relationships would be demonstrated in the TDC and SPD groups, as we did not expect these groups to demonstrate a sufficient range of scores on language measures given their intact language abilities. Indeed, we did not identify relationships between basic auditory processing and verbal abilities in the SPD and TDC groups. We attribute this to a combination of two factors: (a) the differential effect of age on auditory response latency across groups and (b) differences in variance in language abilities across groups. First, there were two participants (one from each of the TDC and SPD groups) who demonstrated late auditory latencies (>270 ms) but did not show impaired scores on language measures (see Figure 4). This is likely attributable to their young ages (8 and 9.5 years, respectively). For example, auditory response latencies have been shown to be age dependent in neurotypical children whereas many individuals with ASD demonstrated later auditory response latencies similar to those of younger typically developing children, suggesting a maturational delay of the auditory response in the ASD group (Edgar et al., 2015). Examination of Figure 4 also suggests that there was indeed insufficient variance in the verbal measures to detect an association between auditory latency and verbal abilities in these groups. In contrast, longer auditory latencies were associated with language impairment in our ASD group, as this was the only group whose range of language abilities spanned from impaired to superior. These associations between cortical auditory response delays and communication skills have been demonstrated in previous auditory evoked response studies of ASD (Oram Cardy et al., 2005a; Russo et al., 2009b; Schmidt et al., 2009; Roberts et al., 2011; Edgar et al., 2013) and children with specific language impairment (Oram Cardy et al., 2008). In contrast, cortical somatosensory delays were not significantly associated with performance on tactile measures, and thus we failed to identify support for Hypothesis 4.

Our final hypothesis (5) was that auditory and somatosensory processing delays are not associated with one another. Indeed, no significant associations were found in these analyses, nor were associations found between cortical processing delays and performance-based processing speed. These findings support our hypothesis that delays in cortical auditory response latency are domain-specific in ASD and are not associated with a generalized processing delay; however, these results must be considered preliminary and need to be replicated before conclusions can be drawn regarding the independence of these processes, particularly considering that support for this hypothesis is based on null findings.

In our previous investigation of white matter in ASD, SPD, and TDC participants, we demonstrated an overlap between ASD and SPD groups with respect to decreased structural connectivity in parieto-occipital tracts; however, reduced connectivity in temporal tracks was restricted to the ASD group (Chang et al., 2014). Likewise, in our recent study examining behavioral measures of auditory and tactile processing, the ASD and SPD groups, while significantly different from TDC participants on a measure of tactile proprioception, only differed from each other by way of greater auditory dysfunction in the ASD group (Demopoulos et al., 2015a). Moreover, this auditory dysfunction was associated with increased communication symptoms in the ASD group, which parallels findings from MEG studies demonstrating associations between auditory response delays/rapid auditory processing deficits and communication impairment (Oram Cardy et al., 2005a; Roberts et al., 2011; Demopoulos et al., 2015b). Results of the present study also supported our hypothesis that cortical auditory response delays are associated with poor communication in ASD participants. Specifically, M200 latencies in the ASD group were negatively associated with verbal skills on the ALI and VCI. Thus, these basic auditory processing deficits may reach a critical threshold in some individuals with ASD, resulting in an adverse impact on higher order processes, including language, from the bottom-up.

While somatosensory response latency was not significantly associated with performance on any of our tactile processing or parent-report measures, prior studies have identified associations between tactile sensory dysfunction and ASD symptomatology (Foss-Feig et al., 2012; Silva et al., 2015a,b). For example, higher tactile detection thresholds in children with ASD were associated with more ASD traits (Tavassoli et al., 2016). In another study examining children longitudinally for tactile response and ASD symptomatology, tactile avoidance at age 9 months was predictive of ASD behavior at 18 months (Mammen et al., 2015). In the present study, tactile response delays distinguished the ASD group from the TDC but not SPD participants, who demonstrated a more intermediate profile. This is consistent with our prior MEG investigation of somatosensory response, in which cortical sensory dysfunction showed a stronger relationship to tactile behavioral group differences than a clinical label of ASD (Marco et al., 2012). Further investigation is required to understand the impact of the somatosensory dysfunction on the developing brain.

### Limitations and future directions

There are several limitations to the present study. First, our sample size was small, which may have limited our ability to detect more modest effects and may have resulted in spurious findings. This is particularly true of the SPD group (*N* = 13), whose results were intermediate of those in the ASD and TDC groups on multiple measures. Replication with a larger sample size is needed to evaluate whether more subtle neurophysiological differences are present in this population. A second limitation was that our sample was restricted to male participants. While this increased homogeneity of our groups, it limits our ability to generalize conclusions to females with ASD and SPD. Future studies examining the auditory and somatosensory cortical response of females in these populations can help elucidate the impact of gender on these variables. Third, seven of our participants were taking medication, and it is unclear what effects these medications may have on performance-based measures or cortical evoked responses. Future research investigating the effects of specific medications on these tasks is necessary to understand the impact they may have on study results. Finally, our measures only focused on somatosensory evoked response from the fingertip on the left hand, whereas somatosensory differences may be more pronounced for other regions of the body in ASD based on clinical presentation for tactile dysfunction. For example, it is possible that difficulty processing oral/laryngeal somatosensory feedback may be associated with speech impairments. Further research is necessary to understand the relationship between somatosensory response delays and functional impairment in ASD. In contrast, this study adds support to several previous findings of associations between auditory processing differences and communication skills in ASD. Longitudinal research in this area is necessary to determine the way these differences emerge and the impact they have during critical periods for language acquisition.

## Conclusions

In addition to replicating findings of abnormalities in cortical auditory and somatosensory response in ASD, this study was the first to demonstrate that these abnormalities are not associated with one another and are not associated with a common generalized processing delay. This study also provided further evidence for the relationship between auditory processing deficits and communication abilities in ASD. Further investigation is warranted to understand the developmental course of this association, as well as the functional impact of somatosensory processing delays on the developing brain in children with ASD and SPD.

## Ethics statement

This study was carried out in accordance with the recommendations of the Committee on Human Research at the University of California-San Francisco (UCSF) with written informed consent from all parents or legal guardians and participants who were of consenting age. Participants who were below consenting age provided written assent for participation in addition to the consent provided by their parent or legal guardian. All subjects gave written informed consent in accordance with the Declaration of Helsinki. The protocol was approved by the UCSF Committee on Human Research.

## Author contributions

CD was responsible for imaging data processing, analysis, interpretation and writing of the manuscript. NY and JT were responsible for imaging data preprocessing. NM performed reliability analysis for peak selection. AB was involved in data management and manuscript review. SD, SH, and AA were involved in study execution, data collection and management. JH was involved in clinical evaluation and classification of participants with SPD. SH and DM supervised collection of MEG data. SN was a mentor to the PI on this project and supervised the design of MEG tasks and processing and analysis of MEG data in addition to providing manuscript review and feedback. EM was the PI of this study and was responsible for the conceptualization, design, and execution of the project as well as supervision of data analysis procedures, interpretation and writing of the manuscript.

### Conflict of interest statement

The authors declare that the research was conducted in the absence of any commercial or financial relationships that could be construed as a potential conflict of interest.

## Footnotes

^1^4D Neuroimaging/Biomagnetic Technologies, I. (n.d.). *Somatosensory Generator*. San Diego, CA.
